# Co-Culture of White Rot Fungi *Pleurotus ostreatus* P5 and *Bacillus amyloliquefaciens* B2: A Strategy to Enhance Lipopeptide Production and Suppress of Fusarium Wilt of Cucumber

**DOI:** 10.3390/jof9111049

**Published:** 2023-10-26

**Authors:** Man Xu, Ying Shi, De-Ling Fan, Yi-Jin Kang, Xin-Li Yan, Hong-Wei Wang

**Affiliations:** 1 Nanjing Institute of Environmental Science, Ministry of Ecology and Environment of China, Nanjing 210042, China; 2 Key Laboratory of Pesticide Environmental Assessment and Pollution Control, Ministry of Ecology and Environmental of China, Nanjing 210042, China

**Keywords:** Fusarium wilt, lipopeptide, iturin, surfactin, RT-qPCR

## Abstract

Fusarium wilt, caused by *Fusarium oxysporum* f. sp. *cucumerinum* (FOC), poses a serious threat to cucumber productivity. Compared to traditional chemical pesticides, biological control strategies have attracted more attention recently owing to their effectiveness against pathogens and their environmental safety. This study investigated the effect of white rot fungi *Pleurotus ostreatus* P5 on the production of cyclic lipopeptides (CLPs) of *Bacillus amyloliquefaciens* B2 and the potential co-culture filtrate of strains B2 and P5 to control cucumber Fusarium wilt. A PCR amplification of CLP genes revealed that *B. amyloliquefaciens* B2 had two antibiotic biosynthesis genes, namely, *itu*A and *srf*, which are involved in iturin A and surfactin synthesis. Liquid chromatography with tandem mass spectrometry (LC-MS/MS) revealed that CLPs derived from strain B2 contained two families, iturin A (C_14_, C_15_) and surfactin (C_12_–C_17_). The co-culture exhibited an enhanced accumulation of iturin A and surfactin compared to the monoculture of strain B2. Furthermore, the gene expressions of *ituA* and *srf* were both significantly upregulated when co-cultured with the fungus compared to monocultures. In an in vitro experiment, the co-culture filtrate and monoculture filtrate of *B. amyloliquefaciens* B2 inhibited mycelial growth by 48.2% and 33.2%, respectively. In a greenhouse experiment, the co-culture filtrate was superior to the monoculture filtrate in controlling cucumber Fusarium wilt disease and in the promotion of plant growth. Co-culture filtrate treatment significantly enhanced the microbial metabolic activity and decreased the abundance of FOC in the rhizosphere soil. These results show that the co-culture of *P. ostreatus* P5 and *B. amyloliquefaciens* B2 has great potential in cucumber Fusarium wilt disease prevention by enhancing the production of bacterial CLPs.

## 1. Introduction

Cucumber is one of the most important vegetables worldwide [1]. However, Fusarium wilt, a destructive soil-borne plant disease caused by the fungal pathogen *Fusarium oxysporum* f. sp. *cucumerinum* (FOC), severely threatens the yield and quality of cucumber [2]. FOC can colonize plant vascular bundles and restrict the transportation of nutrients and water, causing symptoms of leaf yellowing, root rot and death of the whole plant [3]. It has been estimated that Fusarium wilt can cause up to 50% losses in cucumber yield [1,4]. Currently, controlling cucumber wilt disease is mainly dependent on physical and chemical methods [5]. However, physical control, such as soil solarization and soil replacement, is laborious, time consuming, expensive and inefficient [6]. Chemical fungicides cause many negative effects on food safety and can cause environmental pollution [7]. Currently, biological control is regarded as a promising alternative environmentally friendly strategy to cope with soil-borne diseases [6,8].

*Bacillus* spp., ideal biocontrol agents, have been widely used for the biological control of soil-borne plant pathogens [9]. Numerous studies have demonstrated that the main mechanism of *Bacillus* spp. in controlling plant disease is the secretion of bioactive metabolites [10,11]. Among the various antimicrobial compounds produced by *Bacillus* strains, cyclic lipopeptides (CLPs) are considered key compounds for antagonistic activity [12]. Numerous *Bacillus* spp., including *B. amyloliquefaciens*, *B. velezensis*, *B. pumilus*, and *B. subtilis*, have been suggested as valuable biocontrol agents due to the production of CLPs [13,14]. Three main subfamilies of CLPs can generally be distinguished: iturin, surfactin, and fengycin [15]. CLPs share a common cyclic structure comprising a hydrophobic fatty acid chain integrated into a hydrophilic moiety of a cyclized peptide [16]. Additionally, they can maintain high activity under different pH values, high temperatures, and ultraviolet rays [17].

Microbial co-culture (mixed fermentation) typically involves the cultivation of two or more microbes (including bacteria–fungi, bacteria–bacteria, and fungi–fungi) in one culture vessel and is confirmed to be a good strategy to increase the production and variety of secondary metabolites [15,18]. Under microbial co-culture conditions, the microbes can interact with each other and compete for limited living space and nutrients [19]. These interactions between microorganisms will stimulate the expression of gene clusters encoding bioactive secondary metabolites [15]. Li et al. [20] confirmed that the co-culture of *Bacillus subtilis* 22 and *Trichoderma atroviride* SG3403 could produce more antifungal secondary metabolites than the monoculture. Moreover, Ma et al. [21] showed that the mixed fermentation of *B. amyloliquefaciens* and *Trichoderma longibrachiatum* upregulates the expression of biosynthesis genes and the production of CLPs by *B. amyloliquefaciens.* In addition, Akone et al. [19] found that the co-culture of *Bacillus subtilis* with the endophyte fungus *Chaetomium* sp. increased the yield of secondary metabolites by up to 8.3-fold compared with that produced in monocultures.

White rot fungi have been studied due to their strong capacity to degrade a wide range of complex organic pollutants (i.e., polycyclic aromatic hydrocarbons, polychlorinated biphenyls and synthetic textile dyes) by extracellular lignin-mineralizing enzymes (i.e., manganese peroxidase and laccase) and intracellular cytochrome P450 enzymes [22]. In addition, several studies have demonstrated that white rot fungi are also beneficial to the growth of plants by enhancing biological nitrogen fixation [23,24], soil phosphorus mobilization [25] and the amelioration of salinized soils [26]. In a recent study [5], we reported that the combined application of the PGPR *Bacillus amyloliquefaciens* B2 and the plant beneficial white rot fungus *Pleurotus ostreatus* P5 can significantly increase the growth of cucumber under continuous cropping conditions. However, the effect of *P. ostreatus* P5 on the secondary metabolites of *B. amyloliquefaciens* B2 is unclear, especially the production of CLPs. Therefore, the main objectives of this study are as follows: (i) to analyze the CLPs produced by strain B2; (ii) to assess the effect of *P. ostreatus* P5 on CLP synthesis by strain B2; and (iii) to evaluate the efficacy of co-culture filtrates of strains B2 and P5 in controlling FOC under both laboratory and greenhouse conditions.

## 2. Materials and Methods

### 2.1. Microbial Culture and Chemicals

The PGPR *Bacillus amyloliquefaciens* B2 was isolated from the rhizosphere soil of a healthy cucumber plant [5] and stored in Luria–Bertani (LB) medium (10 g L^−1^ peptone, 5 g L^−1^ yeast extract, 10 g L^−1^ NaCl and 20 g L^−1^ agar) at 4 °C. *Pleurotus ostreatus* strain P5 was isolated from spent mushroom (*P. ostreatus*) substrate [27]. The fungal isolate was maintained on potato dextrose agar (PDA) slants at 4 °C. 

*Fusarium oxysporum* f. sp. *cucumerinum* (FOC), a pathogenic fungus, was provided by the Agricultural Culture Collection of China (ACCC; No. 30220) and was stored on PDA slants at 4 °C.

### 2.2. PCR Amplification of B. amyloliquefaciens B2 Lipopeptide Genes

*B. amyloliquefaciens* B2 genomic DNA was extracted using a bacterial genomic DNA extraction kit (Takara, Dalian, China) according to the manufacturer’s instructions. Polymerase chain reaction (PCR) was used to amplify the genes (*ituA*, *srf* and *fen*) involved in lipopeptide (iturin A, surfactin and fengycin) synthesis with the specific primers listed in Table 1. PCR was carried out in a 50 μL reaction volume containing genomic DNA, 1 μL; 10 × PCR buffer, 5.0 μL; 20 mM MgCl_2_, 4 μL; 0.2 mM of each dNTP, 4 μL; 0.5 M of each primer, 1 μL; Taq DNA polymerase (Takara, Dalian, China), 0.3 μL; and ddH_2_O, 33.7 μL. The PCR program consisted of initial denaturation at 95 °C for 4 min; 30 cycles of 95 °C for 35 s, 53 °C for 50 s and 72 °C for 1.5 min; and final extension at 72 °C for 10 min. Each PCR was conducted in triplicate. The amplified products were sequenced by Invitrogen Inc., Shanghai, China. Analysis of the nucleotide sequences was performed with the Blast programs in NCBI GenBank (www.ncbi.nlm.nih.gov, accessed on 18 June 2022). The phylogenetic tree was constructed in the MEGA 7.0 software package using the neighbor-joining method. 

### 2.3. Extraction and Purification of Lipopeptides

The lipopeptides were extracted as described by Ma et al. [10] with slight modifications. Briefly, strain B2 was inoculated into 150 mL of LB medium in a 500 mL Erlenmeyer glass flask and incubated at 30 °C on a rotary shaker at 200 rpm for 72 h. Thereafter, the cell-free supernatant was collected by centrifugation (10,000× *g* at 4 °C for 15 min). The supernatant was adjusted to pH 2.0 with 6 M HCl and kept at 4 °C for 12 h. The precipitate was collected by centrifugation at 10,000× *g* (4 °C) for 20 min and extracted twice with 100% methanol neutralized with sodium hydroxide (NaOH). Samples were dried under N_2_, and the residue was dissolved in 5 mL of 0.01 M, pH 7.4 PBS buffer. Crude extracts were purified through a solid-phase extraction (SPE) C18 cartridge (Agilent, Santa Clara, CA, USA). The SPE column was washed with 10 mL of ddH_2_O, and the lipopeptide compounds were eluted with 2 mL of methanol. The obtained extracts were filtered through a 0.22 μm membrane filter and analyzed by LC-MS/MS.

### 2.4. Identification of Lipopeptides by LC-MS/MS

The extraction products were analyzed by ultra-performance liquid chromatography coupled with a high-resolution hybrid quadrupole Orbitrap mass spectrometer (Q Exactive, Thermo Fisher Scientific, Waltham, MA, USA). A C18 chromatographic column (ZORBAX Eclipse XDB, 2.1 × 150 mm, 3.5 μm; Agilent Technologies, Santa Clara, CA, USA) was used, and the mobile phases were as follows: solvent A, water with 0.1% formic acid; and solvent B, pure acetonitrile. The gradient program was as follows: 0–5 min, 5% B; 5–10 min, 5–30% B; 10–20 min, 30–50% B; 20–40 min, 50–80% B; 40–55 min, 80–95% B; and 55–60 min, 95–5% B. The flow rate was set at 0.3 mL min^−1^. The ionization parameters were as follows: capillary temperature, 330 °C; electrospray voltage, 3.5 kv; tube lens voltage, 35 V; sheath gas flow rate, 40 L/min. Data were acquired in positive ion mode (mass scan range: *m*/*z* 900–1600). The analysis of the data was performed with Xcalibur software version 4.1 (Thermo Scientific, Waltham, MA, USA).

### 2.5. Co-Cultivation of B. amyloliquefaciens B2 and P. ostreatus P5

*B. amyloliquefaciens* B2 was inoculated in LB broth medium at 30 °C with 200 rpm shaking for approximately 12 h. *P. ostreatus* P5 was precultured at 28 °C for 48 h in 100 mL of MYA liquid medium (sucrose 30 g, malt extract 20 g, yeast extract powder 5 g, and water 1000 mL). A liquid co-culture system of strains B2 and P5 was established according to Wang et al. [29] and Ma et al. [21]. In brief, two milliliters of each *B. amyloliquefaciens* B2 and *P. ostreatus* P5 were inoculated in 150 mL of MYA and co-cultured at 30 °C on a rotary shaker at 200 rpm for 2 days. For monoculture, *B. amyloliquefaciens* B2 was grown in 150 mL MYA liquid medium at the same inoculation ratio and incubated at 30 °C for 2 days by shaking at 200 rpm/min. The broth cultures were sampled after 8, 16, 24, 32, 40 and 48 h. The broth cultures were centrifuged at 10,000× *g* for 10 min for further lipopeptide quantification via LC–MS/MS as described above, while bacterial cells were collected for lipopeptide gene expression measurements by RT-qPCR. In addition, after 48 h, the cell density of *B. amyloliquefaciens* B2 was measured by plating 10-fold serial dilutions onto LB agar plates as described by Bartolini et al. [18].

### 2.6. Gene Expression of CLP Biosynthetic Genes

Total RNA was extracted from strain B2 cells using a bacterial RNA kit (OMEGA, Norcross, GA, USA). The concentration of the RNA was determined by a NanoDrop 2000 (Thermo Scientific, Waltham, MA, USA). Total RNA was used as the template for cDNA synthesis using the PrimeScript RT Reagent Kit (TaKaRa, Dalian, China). Specific primers for the expression of the iturin A biosynthetic gene (*itu*-F 5′-AACAGTTCGAAGGCCGGATT-3′, *itu*-R 5′-TTGTCCGCAGCTCACGTAAT-3′) and surfactin biosynthetic gene (*srf*-F 5′-CTTGCCGTTCAAACAGCGAA-3′, *srf*-R 5′-GCATCCCGATCTCCAAGAGG-3′) were designed using Primer 3 Plus software (version 4.0). The RT-qPCR was carried out in an ABI 7500 system (Applied Biosystems, Waltham, MA, USA). The 20 µL reaction volume contained 10 µL 2× SYBR premix Ex Taq (TaKaRa, Dalian, China) with the internal reference dye Rox, 1.0 µL of each gene-specific primer (20 nmol), 1 µL cDNA (100 ng) and 7 µL ddH_2_O. The 2^−ΔΔCT^ method was used to calculate the relative target gene expression level. The *rpsJ* gene (encoding ribosomal protein; *rpsJ*-F 5′-GAAACGGCAAAACGTTCTGG-3′, *rpsJ* -R 5′-GTGTTGGGTTCACAATGTCG-3′), a housekeeping gene in *Bacillus*, was used as the reference gene [30]. All experiments were performed in three independent replicates.

### 2.7. The Effect of Co-Culture Filtrate on FOC Growth

The co-culture or monoculture broth was incubated at 30 °C for 2 days as described in Section 2.5 and filtered through a 0.22 µm cellulose acetate membrane to remove cells before use. Then, 1 mL of co-culture or monoculture filtrates was mixed with 50 mL of PDA (50 °C) at a ratio of 1:50 (*v*/*v*) according to Liu et al. [31]. An FOC mycelial plug (5 mm in diameter) was inoculated at the center of the PDA plate (9 cm in diameter) containing co-culture or monoculture filtrates and cultured at 28 °C for 3 days. The PDA plates without filtrates were used as controls. Each treatment was tested in three replicates. The diameter of the fungal colony was measured after incubation at 28 °C for 7 days, and the inhibition rate of FOC growth was calculated as follows:

Inhibition rate = (C_0_ − C_n_)/(C_0_ − plug diameter) × 100% (1)

where C_0_ is the diameter of the FOC in the control plate, C_n_ is the diameter of the FOC in the filtrate treatment plate, and the plug diameter is 5 mm.

### 2.8. Greenhouse Experiment

Cucumber seeds (*Cucumis sativus* L., JinChun-No.4, Tianjin Cucumber Research Centre, Tianjin, China) were surface sterilized in 0.2% sodium hypochlorite (NaClO) for 3 min and 75% ethanol for 3 min, rinsed four times in sterile water, and then germinated in 9 cm Petri dishes covered with moist filter paper at 30 °C for 36 h. After germination, the cucumber seeds were sown into plastic cups containing 350 g of sterilized nursery soil (121 °C, 30 min). Plant seedlings (one true-leaf stage) were transplanted to plastic pots (10 cm diameter, 15 cm height) with 1.5 kg of soil (6.19 pH, 12.36 g/kg TOC, 1.77 g/kg TN, 0.023 g/kg DOC, 1.69 g/kg TP, 19.47 g/kg TK). The soil was collected from the surface layer (0–15 cm) of a greenhouse located in the Nanjing Institute of Vegetable Science, Nanjing, Jiangsu Province, China (118°46.615′, 31°43.195′). Twenty-four hours before transplantation, the soil in each pot was inoculated with 10 mL of FOC conidial suspension (1.0 × 10^7^ CFU/mL). The culture filtrates were prepared as described in Section 2.5. The three treatments were as follows: (1) CK, irrigated with fresh liquid medium MYA without inoculation; (2) CF-B, irrigated with culture filtrate of strain B2; and (3) CF-BP, irrigated with co-culture filtrate of strains B2 and P5. The fresh MYA medium and culture filtrates were diluted 50-fold, and the cucumber plants were irrigated with 100 mL liquid at days 0, 7, 14 and 21. Each treatment had three blocks with 6 pots for each block. One cucumber seedling was grown in each plastic pot. The experiment was performed in a greenhouse located at the Nanjing Institute of Environmental Science, Nanjing, China. The temperature ranged from 20 to 28 °C, the relative humidity ranged from 62% to 84%, and natural light was provided.

Four weeks after transplanting, the cucumber seedlings were carefully removed from their pots and shaken to remove the soil loosely attached to the plant roots. The soil that tightly adhered to the plant roots was collected and considered cucumber rhizosphere soil. The rhizosphere soil samples were stored at −80 °C for FOC abundance detection and stored at 4 °C to determine soil microbial metabolic activity. Seedling infection by FOC was monitored daily, and the cumulative number of infected cucumber seedlings was also recorded. Disease incidence (DI) was defined as the percentage of infected cucumber seedlings over the total number of plants in each block and was assessed when the disease symptoms appeared (>20% of leaves wilted) [3]. After harvesting, the plant height, root length, and plant dry weight were also measured.

### 2.9. FOC Abundance Detection

Total soil DNA was extracted from 0.25 g of soil using the UltraClean Soil DNA Isolation Kit (Mo Bio Laboratories Inc., Carlsbad, CA, USA). The abundance of FOC in rhizosphere soil was detected using a quantitative real-time PCR assay (ABI 7500, Applied Biosystems, Waltham, MA, USA). The 20 μL reaction mixture contained 10 μL of SYBR Premix *Ex Taq* (Takara, Dalian, China), 1.0 μL of template DNA, 1.0 μL of each FOC-specific primer (FocF3 5′-AAACGAGCCCGCTATTTGAG-3′, FocR7 5′-ATTTCCTCCACATTGCCATG-3′) designed by Lievens et al. [2], 0.4 μL of ROX Reference Dye II (×50, Takara Bio) and 6.6 μL of double-distilled (dd) H_2_O. Sterile water was added as a negative control to replace template DNA. For real-time PCR quantification, standard curves were constructed with a 10-fold dilution series of plasmids (pMD-19T vector, Takara) containing the PCR products from the amplification of strain FOC genomic DNA with the specific primer FocF3/FocR7. All amplifications were carried out in triplicate.

### 2.10. Biolog EcoPlate Analysis

The soil microbial metabolic activity was assessed using a Biolog EcoPlate (Biolog Inc., Hayward, CA, USA) containing 96 wells with 31 different carbon substrates according to Wang et al. [32]. Briefly, 1.0 g soil samples (dry weight) were added to 100 mL 0.85% sterilized NaCl solution and shaken for 30 min (25 °C, 180 rpm). The suspensions were diluted to 10^−3^ with sterilized 0.85% NaCl solution and 150 μL of diluted suspension was inoculated into each well of an EcoPlate. The EcoPlates were incubated in the dark at 25 °C and were measured after 0, 24, 48, 72, 96, 120, 144 and 168 h at 590 nm and 790 nm using a plate reader (BioTek Inc., Winooski, VT, USA). The experiment was carried out in triplicate. The capability of rhizosphere microorganisms to utilize 31 different carbon substrates was calculated by average well-color development (AWCD).

### 2.11. Statistical Analysis

Comparisons between the means of two independent groups were carried out with Student’s *t*-test, and multiple comparisons were performed with one-way analysis of variance (ANOVA) and Duncan’s test using SPSS software (version 17.0; SPSS Inc., Chicago, IL, USA). *p* < 0.05 was considered significant.

## 3. Results

### 3.1. Identification of Lipopeptide Biosynthetic Genes from B. amyloliquefaciens B2

**Figure 1:**
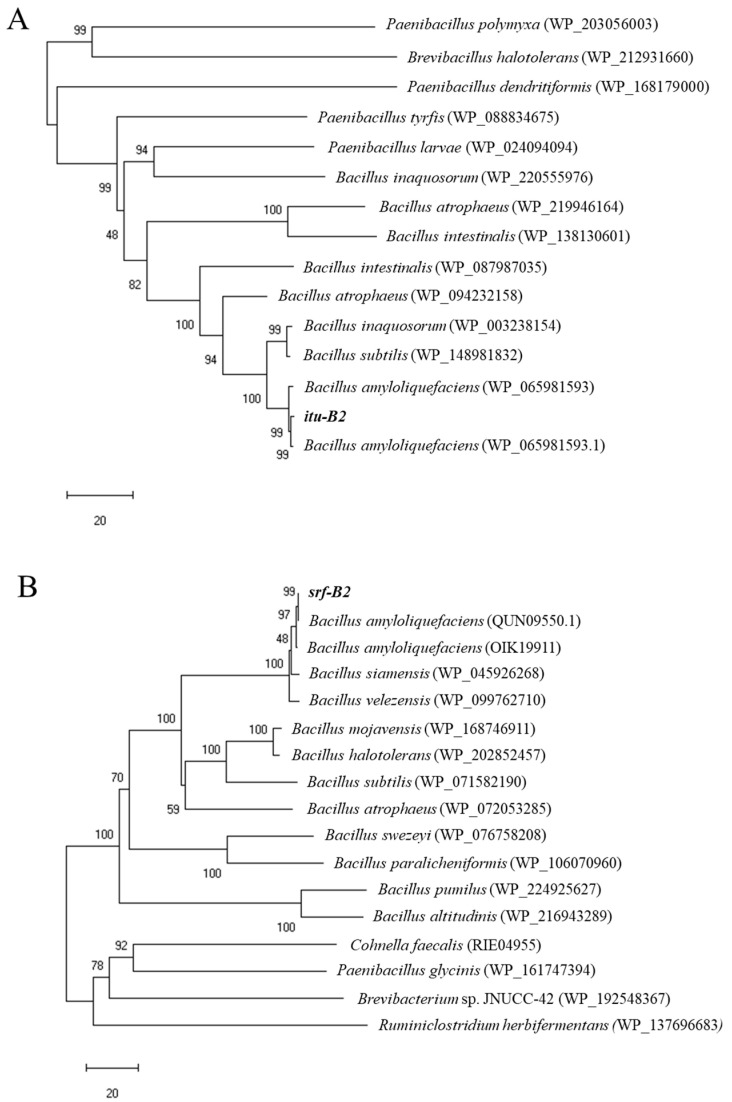
Phylogenetic analysis of amino acid sequences of iturin A synthetase (**A**) and surfactin synthase (**B**). The trees were constructed by the neighbor-joining method. The reliability of the inferred trees was tested by bootstrap analysis using 1000 replications.

The *itu*A (iturin A synthase) and *srf* (surfactin synthase) genes were amplified in the *B. amyloliquefaciens* B2 genome but not those encoding *fen* (fengycin synthase) (Table 2). The iturin A synthetase and surfactin synthase genes were designated as *itu*-B2 (1145 bp) and *srf*-B2 (1167 bp), respectively (Supplementary Figure S1). The deduced amino acid sequence of the *itu*-B2 protein showed a maximum homology of 98.7% with the known iturin A synthase of *B. amyloliquefaciens* (WP_065981593.1) (Figure 1A). The deduced amino acid sequence of the *srf*-B2 protein showed a maximum homology of 99.7% with the known surfactin synthase of *B. amyloliquefaciens* (QUN09550.1) (Figure 1B).

### 3.2. Identification of CLPs Produced by B. amyloliquefaciens B2

**Figure 2:**
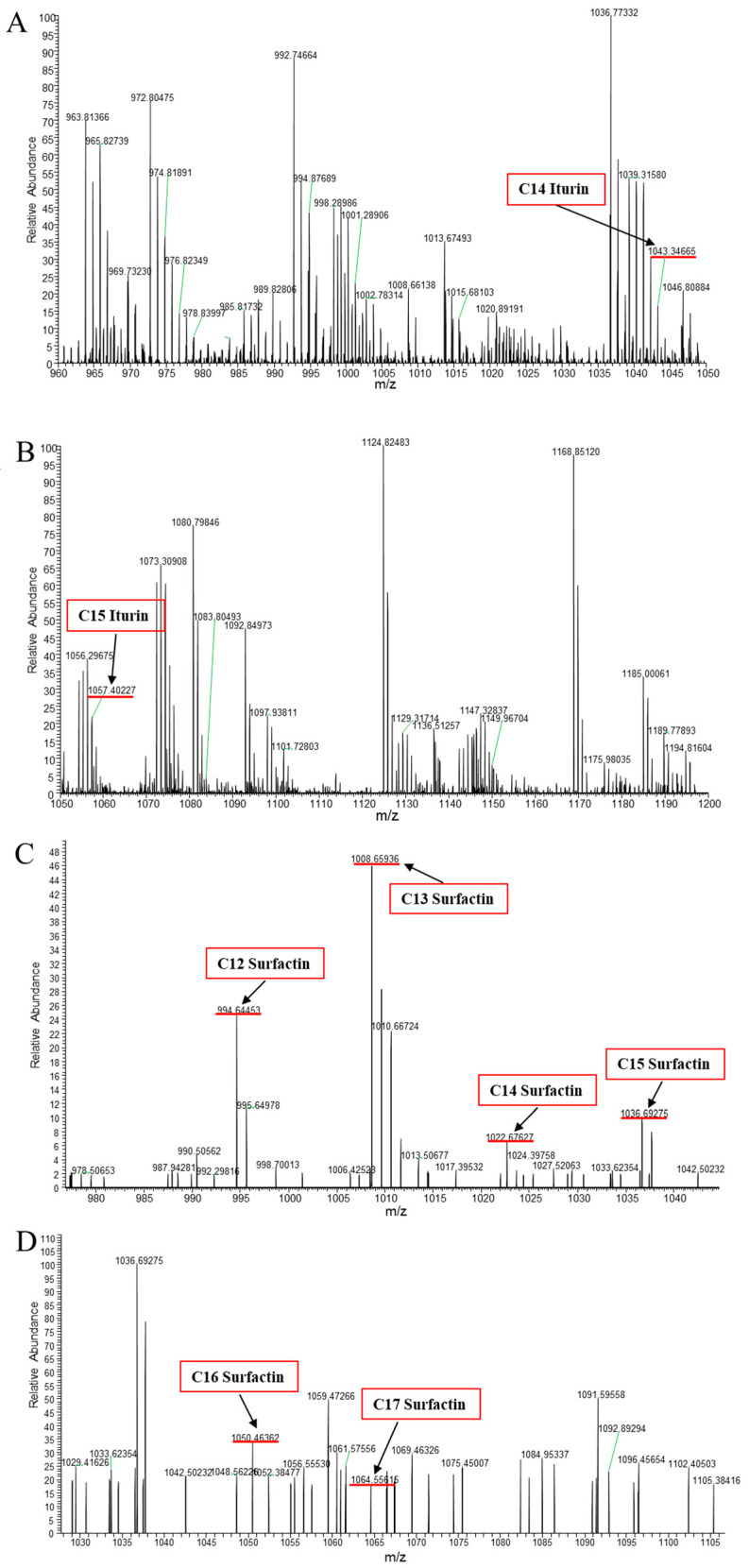
LC-MS/MS for the lipopeptide antibiotics extracted from *Bacillus amyloliquefaciens* B2. (**A**) Spectra of inturin A in the range of *m*/*z* = 960–1050 Da; (**B**) spectra of inturin in the range of *m*/*z* = 1050–1200 Da; (**C**) spectra of surfactin in the range of *m*/*z* = 1050–1200 Da; (**D**) spectra of surfactin in the range of *m*/*z* = 1050–1200 Da.

LC-MS/MS analysis showed the production of the lipopeptides iturin A and surfactin from *B. amyloliquefaciens* B2 (Figure 2, Table 3). As shown in Figure 2, the protonated molecular ion ([M + H]^+^) peaks of the iturin A homologues (C_14_ and C_15_) were detected at *m*/*z* 1043.34665 and 1057.40227 Da, and the peaks with differences of 14 Da may correspond to the molecular weight of one CH_2_ group. In addition, peaks at *m*/*z* 994.64453, 1008.65936, 1022.67627, 1036.69275, 1050.46362 and 1064.55615 Da may be assigned as the protonated molecular ions ([M + H]^+^) of the C_12_, C_13_, C_14_, C_15_, C_16_ and C_17_ homologues of surfactin (Figure 2, Table 3).

### 3.3. Effect of P. ostreatus P5 on the Production of CLPs and Transcription of Their Encoding Genes by B. amyloliquefaciens B2

**Figure 3:**
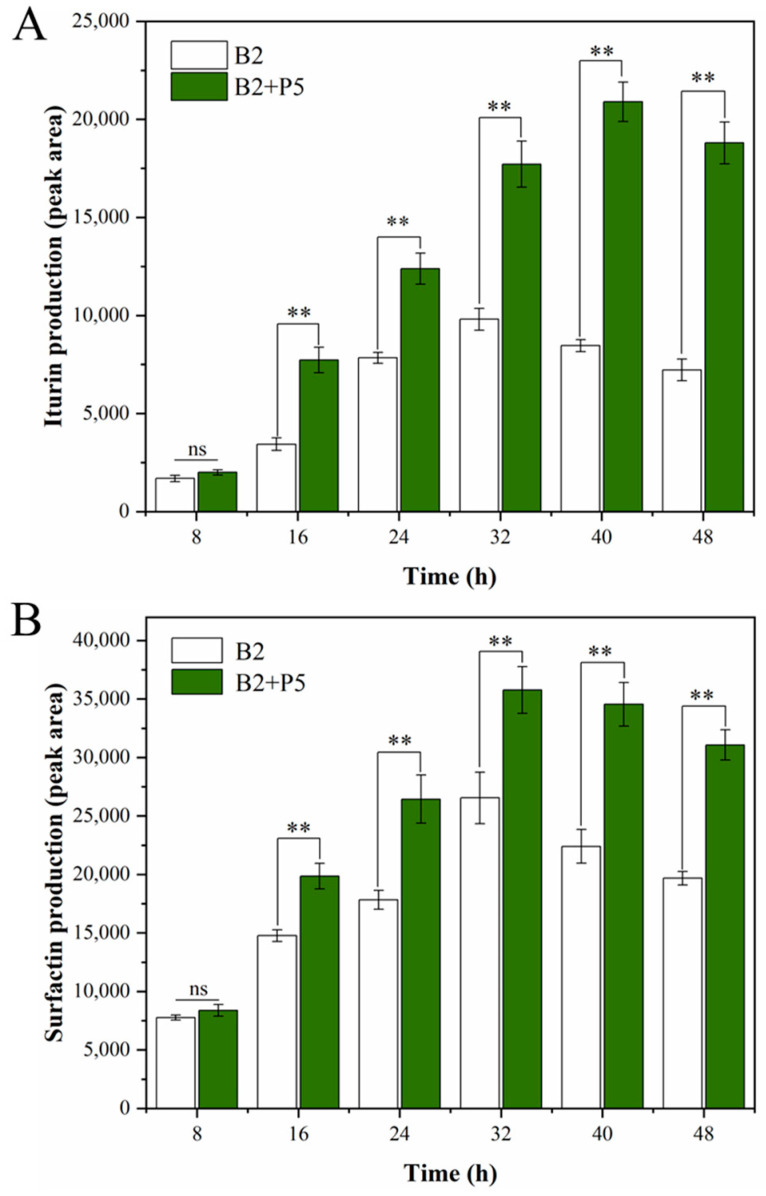
Impact of the *Pleurotus ostreatus* P5 on the production of iturin A (**A**) and surfactin (**B**) by *Bacillus amyloliquefaciens* B2. Statistical significance was calculated using *t*-test (ns, no significant difference (*p* > 0.05); **, *p* < 0.01).

CLPs produced by strain *B. amyloliquefaciens* B2 in monoculture or in co-culture with *P. ostreatus* P5 were extracted and quantified at 8 h intervals. As shown in Figure 3, increases in iturin A and surfactin production were detected in the presence of *P. ostreatus* P5. The production of iturin A and surfactin reached the highest levels at 40 h and 32 h, respectively, under the co-culture conditions. Moreover, there was no significant difference in the cell density of *B. amyloliquefaciens* B2 under monoculture and co-culture conditions after 48 h (Supplementary Figure S2).

**Figure 4:**
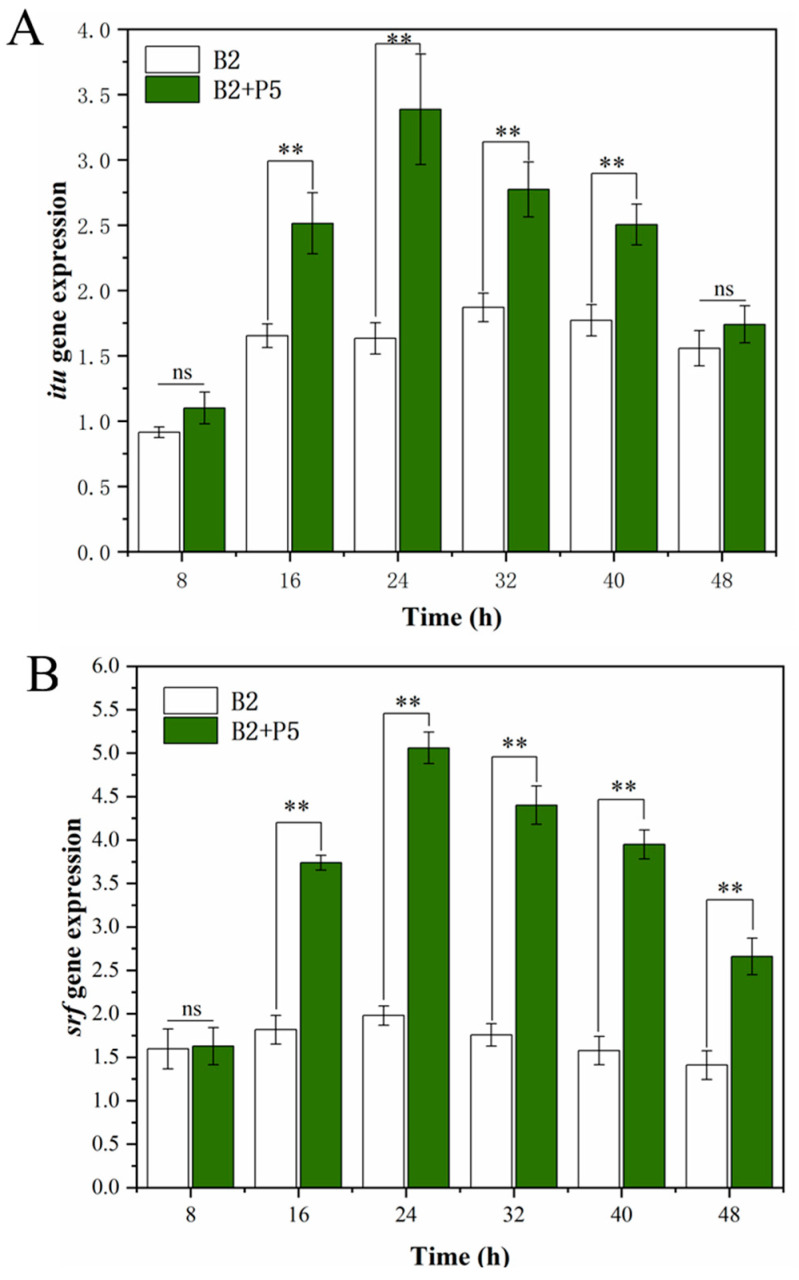
Impact of the *Pleurotus ostreatus* P5 on the *ituA* (**A**) and *srf* (**B**) gene expression by *Bacillus amyloliquefaciens* B2. Statistical significance was calculated using *t*-test (ns, no significant difference (*p* > 0.05); **, *p* < 0.01).

The dynamics of iturin A synthase and surfactin synthase gene expression levels within 48 h were detected, and the results are shown in Figure 4. When *B. amyloliquefaciens* B2 was grown in the presence of *P. ostreatus* P5, the mRNA expression level of iturin A synthetase increased to the highest level at 24 h, which was 2.1-fold higher than that in the monoculture (Figure 4A). Similar to iturin A synthase, expression of the surfactin synthase gene in *B. amyloliquefaciens* B2 confronted with *P. ostreatus* P5 increased rapidly to a peak at 24 h, which was 2.6-fold higher than that of monocultivation of strain B2 (Figure 4B).

### 3.4. Inhibition of FOC Growth by Co-Culture Filtrate

**Figure 5:**
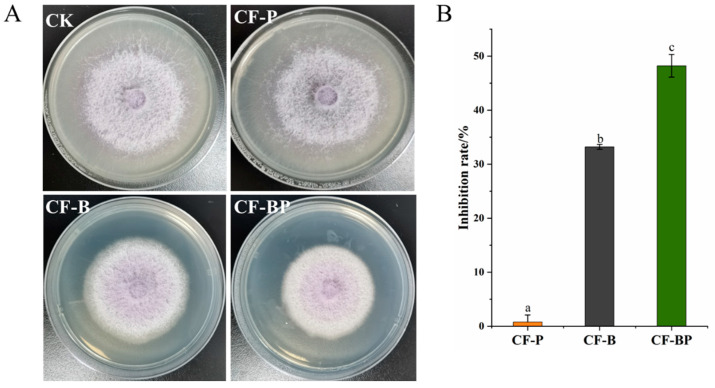
The effect of the co-culture filtrate of *Bacillus amyloliquefaciens* B2 and *Pleurotus ostreatus* P5 on FOC growth. (**A**) Growth inhibition of the co-culture filtrate on FOC. (**B**) Inhibition rates of the co-culture filtrate on FOC. CF-P, culture filtrate of strain P5. CF-B, culture filtrate of strain B2. CF-BP, co-culture filtrate of strains B2 and P5. Different letters above the bars indicate statistically significant differences according to Duncan’s test (*p* < 0.05).

To evaluate the inhibitory effect of the co-culture filtrate of *B. amyloliquefaciens* B2 and *P. ostreatus* P5 on FOC growth, the filtrate was mixed with PDA medium and tested, and the antimicrobial effect of the co-culture filtrate reached 48.2%, which was significantly (*p* < 0.001) higher than that of the monoculture filtrate (Figure 5). The inhibitory rates of the monoculture filtrate of strain B2 were 33.2%, while the filtrate culture of *P. ostreatus* P5 had no inhibitory effect (Figure 5).

### 3.5. Effect of Co-Culture Filtrate against Cucumber Fusarium Wilt

**Figure 6:**
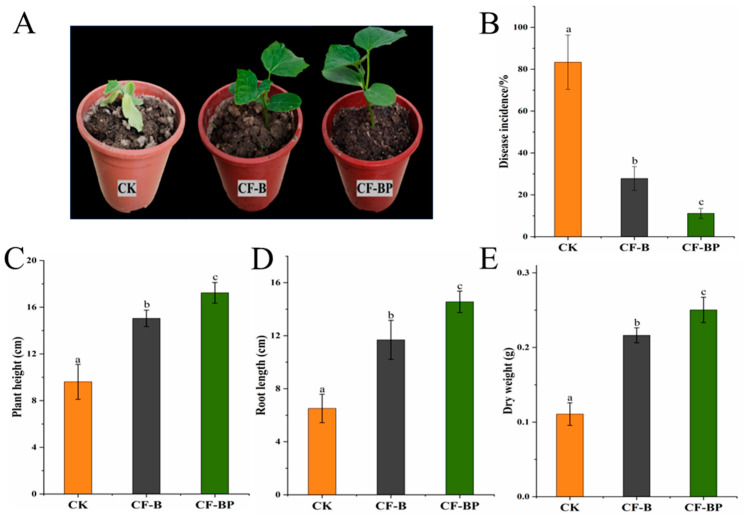
Plant Fusarium wilt suppression and growth promotion by culture filtrates. (**A**) The growth and disease phenotype of cucumber plant. (**B**) Disease incidence. (**C**) Plant height. (**D**) Root length. (**E**) Plant dry weight. CF-B, culture filtrate of strain B2. CF-BP, co-culture filtrate of strains B2 and P5. Different letters above the bars indicate statistically significant differences according to Duncan’s test (*p* < 0.05).

In this study, we investigated the potential roles of the co-culture filtrate of strains B2 and P5 in suppressing Fusarium wilt and promoting plant growth in cucumber. After 4 weeks of cucumber growth, the leaves of the cucumber plants in the CK treatment turned yellowing and were withering (Figure 6A). The CF-BP treatment was barely affected by FOC infection (Figure 6A). Using the culture filtrates significantly suppressed Fusarium wilt disease incidence in cucumber, which decreased from 83.3% in the untreated control to 27.8% in the monoculture treatment and 11.1% in the co-culture filtrate treatment (Figure 6B). The co-culture filtrate of strains B2 and P5 increased the plant height, root length, and plant dry weight by 79.3%, 138.9%, and 126.1% compared to CK, respectively, and by 14.5%, 24.6%, and 15.7% compared to the monoculture filtrate of strain B2 (Figure 6C–E).

### 3.6. Effect of Culture Filtrate on the Abundances of Rhizosphere FOC and Microbial Metabolic Activity

**Figure 7:**
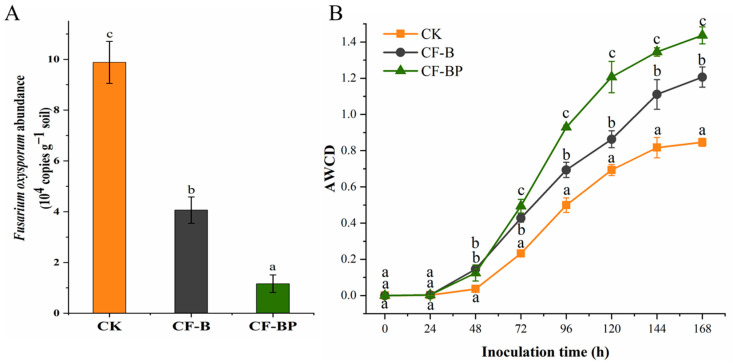
Effect of culture filtrates on the abundances of *Fusarium oxysporum* (**A**) and microbial metabolic activities (**B**) in the cucumber plant rhizosphere soil. CF-B, culture filtrate of strain B2. CF-BP, co-culture filtrate of strains B2 and P5. Different letters above the bars indicate statistically significant differences according to Duncan’s test (*p* < 0.05).

The abundances of FOC in rhizosphere soil were significantly influenced by fermentation liquid amendment. Figure 7A shows that the soil sample amended with co-culture filtrate of strains B2 + P5 contained the lowest copy numbers of FOC compared to the other treatments. The CF-B and CF-BP treatments significantly reduced the copy numbers of FOC by 58.9 and 88.3% (on average) compared to the control, respectively (Figure 7A). Soil microbial metabolic activity was assessed through the measurement of average well color development (AWCD) in a Biolog EcoPlate (Figure 7B). The AWCD values were higher in the culture filtrate-treated soils than in CK soil. The AWCD in the CF-B and CF-BF treatments increased by 42.6 and 69.8%, respectively, compared to those of the control (Figure 7B).

## 4. Discussion

*Bacillus* strains have previously demonstrated biocontrol ability against Fusarium wilt in cucumber, banana, and tomato [33]. The production of antibiotic metabolites, especially CLPs, is recognized as the most important mechanism contributing to their biocontrol potential against soil-borne plant pathogens [10]. PCR analysis using specific primers performed in the current study confirmed that strain B2 harbors biocontrol genes encoding the synthetases of lipopeptides iturin A and surfactin. A phylogenetic tree constructed based on the protein sequences of the *ituA* and *srf* genes of strain B2 also showed clear homology with the target proteins. Similarly, Nanjundan et al. [34] documented the presence of genes encoding iturin A and surfactin lipopeptides in *B. amyloliquefaciens* SR1. Moreover, Ding et al. [6] detected genes involved in the synthesis of iturin A, surfactin and fengycin in *B. mojavensis* YL-RY0310, while another study reported *B. subtilis* MK3 with only a fengycin synthetase gene [35]. 

Several species of *Bacillus* are known to produce different lipopeptides [34]. Three families of lipopeptides viz. Iturin, surfactin, and fengycin are primarily produced by *Bacillus* spp. [13]. In this study, LC-MS/MS analysis revealed that the CLPs produced by strain B2 comprise a mixture of iturin A and surfactin, which is consistent with the biosynthesis genes detected by PCR assay. The iturin A produced is composed of a lipid chain of length C_14_ to C_15_, while the lipid chain length of surfactin ranges from C_12_ to C_17_. According to Malakar et al. [36], the CLPs produced by *B. licheniformis* strain SCV1 were C_13_, C_14_, C_15_, C_16_ and C_17_ iturin homologues and C_12_, C_13_, C_14_, C_15_ and C_16_ surfactin homologues. In addition, Zhang et al. [37] only detected lipopeptides fengycin (C_15_–C_17_) in the culture filtrate of *B. atrophaeus* CAB-1, while no detectable surfactin or iturin compounds were found. Thus, our study showed the structurally diverse homologues of iturin A and surfactin mixture in the CLPs produced by *B. amyloliquefaciens* B2.

The co-cultivation strategy has proven to be a potential technology to improve the metabolic production of microbes by enhancing the expression levels of target genes compared to monoculture [15]. In the present study, the co-culture of *B. amyloliquefaciens* B2 and *P. ostreatus* P5 induced an increase in the amounts of iturin A and surfactin secreted by strain B2. These findings are consistent with several studies suggesting that CLPs produced by *Bacillus* spp. were affected when co-cultivated with various fungi [15,16,38] and provide further evidence of the regulation of CLPs produced by *Bacillus* spp. through direct interactions. For example, there was a twofold increase in the amounts of iturin A and surfactin produced by *B. subtilis* IM13 when confronted with *F. oxysporum* [39]. Similar results showed an overproduction of iturin A and fengycin when *B. subtilis* 98S was cocultured with *Fusarium* and *Pythium* compared to the monoculture but not in the presence of fungal *Botrytis* [40]. In contrast, decreases in surfactin production were detected when *B. subtilis* was co-cultured with *Aspergillus niger* compared with the monoculture of *B. subtilis* [41]. Additionally, after 48 h, the cell density of *B. amyloliquefaciens* B2 was not significantly changed during co-culture compared to monoculture. Similar results were reported previously for the co-culture of *Eurotium amstelodami* and *Bacillus licheniformis* [29].

The gene expression assay based on RT-qPCR showed that the iturin A and surfactin synthase genes of strain B2 were upregulated under the co-culture condition. This is in line with previous reports that the expression levels of surfactin and iturin biosynthesis genes in *B. amyloliquefaciens* Am1 were higher during the co-culture interaction with *R. solanacearum* compared with those in strain Am1 monoculture [8]. The iturin A and fengycin genes of *B. amyloliquefaciens EZ1509* were upregulated by the presence of the fungus *Sclerotinia sclerotiorum* [28]. However, limited research is available on the bacterial gene regulatory mechanisms of the production of CLPs under co-culture conditions. Recently, it was demonstrated that some chemical and biotic signals emitted by the fungal mycelium could be perceived by bacterial cells, which in turn modulates antibiotic production [40]. Bartolini et al. [18] also revealed that the *fungus F. verticillioides* could activate the stress-responsive sigma B transcription factor of *B. subtilis*, which subsequently increased the expression of the operon responsible for the biosynthesis of surfactin. Further studies are still needed to identify the signaling molecules triggering the expression of CLPs released by *P. ostreatus* P5. As predicted, the maximal expression levels of iturin A synthase and surfactin synthase gene were both detected before the appearance of maximal production of iturin A and surfactin. These differences may be because it still needs some time to translate and modify enzyme protein after gene expression. Kulimushi et al. [15] also showed that the appearance of a maximal expression of fengycin synthase gene was earlier than that of the maximal fengycin production.

In the present study, co-culture and pure bacterial culture filtrates both significantly inhibited the mycelial growth of FOC (Figure 4). These results are also in agreement with those observed in several studies [42]. For example, Solanki et al. [11] reported that the culture filtrate of *B. amyloliquefaciens* MB101 inhibited the hyphal growth of *Rhizoctonia solani* at a rate of 77.6%. Additionally, a previous study demonstrated that filtrate from *B. velezensis* AR1 reduced the hyphal growth of *Glomerella cingulata* by 50% [7]. *Bacillus* mostly inhibit fungal pathogens via the secretion of CLP metabolites, and it has been shown that mutant *Bacillus* strains deficient in CLPs do not possess antifungal activity [43,44]. The main mechanism of iturin lipopeptides against fungi is the increase in cytoplasmic membrane permeability and cause of cell structure disruption. Furthermore, surfactin may interact with phospholipid acyl chains, resulting in abnormal mycelial growth and the lysis of intracellular organelles [45,46]. Iturin A exhibits a synergistic effect with surfactin with respect to antifungal activity [12,47]. Therefore, it can be speculated that the higher antifungal activity of co-culture filtrate than that of the monoculture filtrate is likely associated with the increased production of CLPs [44].

Pot experiments revealed that culture filtrates possess significant potential in suppressing cucumber Fusarium wilt and promoting plant growth. Similarly, Chen et al. [48] observed that the biocontrol efficiency of the culture filtrate of *B. velezensis* FJAT-46737 against tomato bacterial wilt was 82.0%. We found that the biological control effect of the filtrate from co-culture was better. This is in agreement with the report that the mixed culture fermentation of *B. amyloliquefaciens* and *Trichoderma longibrachiatum* can effectively control Fusarium wilt in tomato and had a better control effect than the pure bacterial culture [21]. Moreover, Sarwar et al. [17] and Pal et al. [49] also reported that CLPs from culture filtrates could improve the growth of rice and maize seedlings and suppress plant disease. On the other hand, several studies have demonstrated the CLP molecules not only exhibit excellent antifungal activity but also act as bioactive compounds to induce plant defense in strawberry [50], tomato [33] and rice [51]. 

Soil biological parameters, especially soil microbial metabolic activity, are considered core indicators of agricultural soil quality and are also important indicators in the restoration process of agroecosystems [5]. The loss of microbial catabolic activity or of the diversity of carbon source use is regarded as a warning of diminishing agricultural soil health [52]. Several reports have indicated that the microbial function in carbon source utilization decreased significantly in the rhizosphere soil of diseased plants with vascular wilting symptoms [53]. Our results showed that compared with the CK treatment, the AWCD values (representing microbial metabolic activity) were significantly increased in the CF-BF treatment. These findings were in accordance with those reported by Másmela-Mendoza et al. [53], where the application of the culture filtrate of *B. velezensis* Bs006 increased microbial metabolic activity in the wilt-diseased tomato plant rhizosphere. Root exudates, which are secreted at the soil–root interface, have been considered the most important factor affecting microbial metabolic function in rhizosphere soil [52]. The increase in AWCD values in the culture filtrate application treatment might be due to changing the composition and amount of root exudates caused by the enhanced growth of cucumber.

## 5. Conclusions

In this study, PCR amplification analysis of CLP genes demonstrated that *B. amyloliquefaciens* B2 harbors genes involved in iturin A and surfactin biosynthesis. Furthermore, two antifungal lipopeptides, iturin A and surfactin, were detected in strain B2 culture extracts by LC-MS/MS. Compared with monoculture, the co-culture conditions can significantly induce strain B2 to produce more lipopeptides and upregulate the expression of related genes. The co-culture filtrate exhibited strong antifungal activity against FOC by inhibiting mycelial growth. The co-culture filtrates of *B. amyloliquefaciens* B2 and *P. ostreatus* P5 promoted cucumber growth and protected plants against FOC under greenhouse conditions. In addition, the co-culture filtrates significantly improved the soil microbial metabolic function and reduced the abundance of FOC. Overall, this study indicates that the co-culture system with strains B2 and P5 provides an effective strategy for the biocontrol of Fusarium wilt disease. Further studies are needed to optimize the co-culture medium to increase the production of CLPs and reduce the cost of fermentation.

## Figures and Tables

**Table 1:** The specific primers for detection of lipopeptide-related genes in *Bacillus amyloliquefaciens* B2.

Lipopeptides	Gene	Primer Sequence (5′-3′)	Reference
Iturin A	*ituA*	ACCTCACCTTGATCGGCTATAC	[28]
		TGGTGGGCGAAAGAAGTTTATG	
Surfactin	*srf*	CGGTGATCTTGCGAAGCTTTAT	[28]
		CGCTTTCGTTCTGCCATTCT	
Fengycin	*fen*	CGGCCATTCGCTCATCTTTAT	[28]
		GTTTCCGCTTCATCAGTCTCTTC	

**Table 2:** The PCR amplification results of CLPs genes in *Bacillus amyloliquefaciens* B2.

Material	Gene	Strain B2
Iturin A	*ituA*	+
Surfactin	*srf*	+
Fengycin	*fen*	−

+: positive; −: negative.

**Table 3:** Calculated mass values of M + H^+^ ions corresponding to identified isoforms of iturin A and surfactins in culture extracts from *Bacillus amyloliquefaciens* B2.

Lipopeptides	Fatty Acid Chain	Molecular Formula	Calculated (*m*/*z*)	Reference
			[M + H]^+^	
Iturin A	C_14_	C_48_H_74_N_12_O_14_	1043.34665	[13]
	C_15_	C_49_H_76_N_12_O_14_	1057.40227	[13]
Surfactin	C_12_	C_50_H_87_N_7_O_13_	994.64453	[14]
	C_13_	C_51_H_89_N_7_O_13_	1008.65936	[14]
	C_14_	C_52_H_91_N_7_O_13_	1022.67627	[14]
	C_15_	C_53_H_93_N_7_O_13_	1036.69275	[14]
	C_16_	C_54_H_95_N_7_O_13_	1050.46362	[9]
	C_17_	C_55_H_97_N_7_O_13_	1064.55615	[9]

## Data Availability

Data supporting reported results are presented in this paper along with its Supplementary Materials.

## Supplementary Materials

The following supporting information can be downloaded at: https://www.mdpi.com/article/10.3390/jof9111049/s1, Figure S1: The partial nucleotide sequences of iturin synthetase gene (A) and surfactin synthase gene (B) obtained in our study; Figure S2: Comparison of cell density of *Bacillus amyloliquefaciens* B2 under different culture conditions.
