# Supplementary material for: Co-Culture of White Rot Fungi Pleurotus ostreatus P5 and Bacillus amyloliquefaciens B2: A Strategy to Enhance Lipopeptide Production and Suppress of Fusarium Wilt of Cucumber
Source: J Fungi (Basel). 2023 Oct 26;9(11):1049. doi: 10.3390/jof9111049 (PMC10672132; doi:10.3390/jof9111049)
Supplement: Supplementary file 1 [file jof-09-01049-s001.zip › jof-2637969-supplementary.pdf]

Supplementary Information

Co-Culture of White Rot Fungi *Pleurotus ostreatus* P5 and *Bacillus amyloliquefaciens* B2: A Strategy to Enhance Lipopeptide Production and Suppress of Fusarium Wilt of Cucumber

Man Xu [1,2,†], Ying Shi [1,2,†], De-Ling Fan [1,2], Yi-Jin Kang [1,2], Xin-Li Yan [1,2] and Hong-Wei Wang [1,2,*]

[1] Nanjing Institute of Environmental Science, Ministry of Ecology and Environment of China, Nanjing 210042, China; xuman@nies.org (M.X.); shiying@nies.org (Y.S.); fdl@nies.org (D.-L.F.); 13121010125@163.com (Y.-J.K.); nailaixykb8244@163.com (X.-L.Y.)
[2] Key Laboratory of Pesticide Environmental Assessment and Pollution Control, Ministry of Ecology and Environmental of China, Nanjing 210042, China
* Correspondence: wanghongwei@nies.org
† These authors contributed equally to this work.

A
CCGTTGTATTCGTCAATCTCTTTTGATCTGACGGTGACTTCTATCTTCACA
CCGCTTGTCACGGGCAATACGATCATCGTCTACGACGGTGAGGATAAAA
CAGCACTGCTTGCCTCGATTGTGCAGGACCCGAGGGTGGATATCATTAA
ATTGACCCCGGCCCATTTGCAGGTTTTGAAAGAAATGAACCTCGCCGAT
AAAACGGCTGTCCGAAGAATGATTGTGGGCGGGGAGAATCTAAGCACC
CGGTTAGCCCAAAGCATTCATGAACAGTTCGAAGGCCGGATTGAAATAT
GCAATGAGTACGGGCCGACTGAAACCGTTGTCGGCTGCATGATTTACCG
TTACGATGCCGTAAAAGACAGGCGGGAATCGGTACCGATCGGAACGGC
TGCAGCAAACATGAGCATTTATGTTCTTGATGAGAATAGGAAACCCGTG
CCGATCGGTGTCCCTGGGGAAATCTATATCAGCGGCGCCGGCGTGGCAA
GAGGATATTTAAACCGGCCGGAATTGACGGCAGAGAAATTTGTCGATGA
CCCTTTTGAACCCGCCGGAGCCAAGATGTATAAAACCGGAGATTTGGCG
AAGTGGCTTGCTGACGGAAACATCGAATATGCGGGAAGAATAGATGAA
CAAGTAAAAATCAGAGGCTATCGTATTGAATTAGGAGAGATCGAGGCGG
CACTGCACCAAGAAGAAGCGATCAAGGAAGCGGTCGTGACAGCCAGA
GAAGATGTCCACGGGTTCAAACAATTGTGTGCATATTACGTGAGCTGCG
GACAAATAACGGTATCCAAGCTTCGGAAACAATTGTCTCAAACTCTGGC
AAGTTATATGATTCCGGCTTATTTTATCGAGATGGATGAAATGCCTTTAAC
CTCCAACGGAAAAATAAACAGAAAAGAGCTGCCGGCTCCGGATTTCGG
CCTGCAGGACAGAGCAGAGTATAAGGCTCCCCGAACGAAAGCGGAAG
AAATATTAGTATCTGTCTGGGAATCGGTATTAGGCGCAGAGAACATCGGT
ATTCTGGATAATTTCTTTGATCTCGGCGGAGATTCGATTAAATCAATTCAA
GTGTCATCCAGATTAAAACAAAGCGGATACAAAATGGAAATCAAGGACT
TGTTTCAATATGCAACGAT

B
CGCGGTCATATTTCTCTCATTGACGACGAGAAGCTTCGTCCCGCTGTCTT
CCAGAGAGTAGCGGATTCTCTCTTCAGGCGTCTCCGGATCAATCGGGAG
AAACGCAGCCCCGGCTTTCATGACACCGAGAATCGCGGTAATTAAGTCA
GCCGAACGATCCATCATAATGGCAACGATGTCCTCGCTGCCCGCTCCGC
AGGCCTGTAGGCGGCGCGCAAACCGGTTCGACTGTTCATCAAGCTCCC
GATATGTCAGACTTGTTCCGCCCGCAACCGCCGTGAGATCAGGCGTTTT
TTGCGCTTGCCGTTCAAACAGCGAAACAAAAGTGCCTTCAGGCAGGTC
CGCTTCCGTACGTTTTTCATCAGAAAGGAGCGCTTCCCGCTCAGAAGCA
GACAACAGGCTTGCATCAGCCAGCTGAATATCCGGATTTTCAGCGCAGG
CTTTAATCAGATTGACAAAATGAATACTCCAGCGGCGGATTGTTTCGTCC
GCAAATAAGGCTTTCGCATAATCAAAGCTCAATCCGACCTCTTCTCCGC
GTTCAGCCGCTTCTAAAGAAAGATCAAATTTAGCGACATGATGATGCAT
GGAATATGAAGAAATCTGCAAATCACCGAGCCGCAGAGACGGGATCTC
CATGTTCTGCATATTAAATAACACGCTGAACAGAGGACTCCGGCTTGTG
TCCCTCGTTAACGGCAGTTTTTCAATTAATTCTTCAAGCGGATAGCTTTG
ATGCTCAAAAGCTTGAAGACTCGCCGTTTTCACCTCGTCCAGAAATTGC
TTGAACGTTTTTTTGCCGGCAGGATGCGACCGAAGCGCAACCGTGTTGA
CAAACATGCCCGGCACCTCTTGGAGATCGGGATGCTGTCTTCCGGCTGC
CGGAGATCCGACTATGATATCTTCCTGCCCTGAATATTTAGAGAGCAGCA
CTTGAAAAGCGGCTAACAAGAACATGTACATCGTCGTATCCGTATCAGC
CAGCAATGTCTTAATCTGCGCTGTTACTTGCTTATCAAGTCCGAAAATGA
CGCGTTCCCCTTCAAAACTGCGCTCGGCAGGACGCGGGAAATCAAGCG
GCAGGTCCAGCACCGGCAGATCGCCTTTGAACGTATCGAGCCAGTAAG
CTTCGTGCTGATCCAGATTTGCTTTTTCACGCTGCCAGAC

Figure S1. The partial nucleotide sequences of iturin synthetase gene (**A**) and surfactin synthase gene (**B**) obtained in our study.

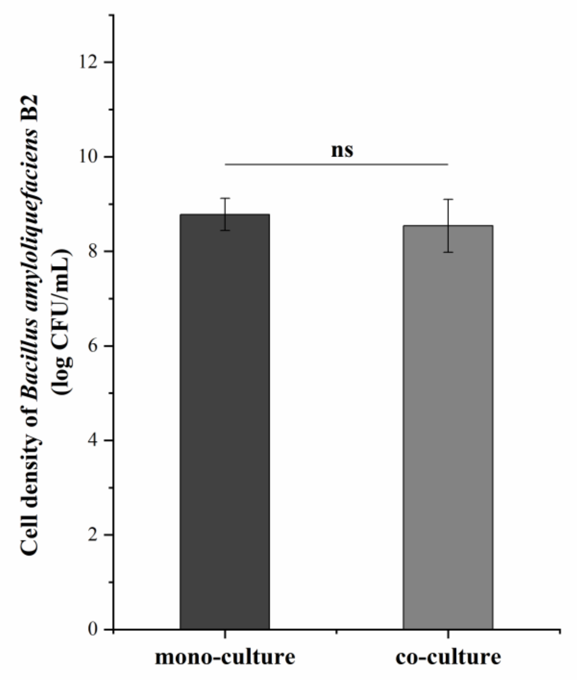

Figure S2. Comparison of cell density of *Bacillus amyloliquefaciens* B2 under different culture conditions. Statistical significance was calculated using t-test where "ns" means no significant difference ($p > 0.05$).
